# Molecular Epidemiology of Dengue Viruses in Lao People’s Democratic Republic, 2020–2023

**DOI:** 10.3390/microorganisms13020318

**Published:** 2025-02-01

**Authors:** Cécile Troupin, Kedkeo Intavong, Somphavanh Somlor, Souksakhone Viengphouthong, Sitsana Keosenhom, Thep Aksone Chindavong, Phaithong Bounmany, Longthor Vachouaxiong, Thonglakhone Xaybounsou, Chittaphone Vanhnollat, Phonepadith Khattignavong, Darouny Phonekeo, Bouaphanh Khamphaphongphane, Phonepadith Xangsayarath, Vincent Lacoste, Philippe Buchy, Gary Wong

**Affiliations:** 1 Virology Laboratory, Institut Pasteur du Laos, Vientiane 01030, Laos; c.troupin@pasteur.la (C.T.); k.intavong@pasteur.la (K.I.); s.somlor@pasteur.la (S.S.); s.viengphouthong@pasteur.la (S.V.); s.keosenhom@pasteur.la (S.K.); t.chindavong@pasteur.la (T.A.C.); p.bounmany@pasteur.la (P.B.); l.vachouaxiong@pasteur.la (L.V.); xbs.thonglakhone@gmail.com (T.X.); c.vanhnollat@pasteur.la (C.V.); vincent.lacoste@pasteur.fr (V.L.); 2 Parasitology Laboratory, Institut Pasteur du Laos, Vientiane 01030, Laos; p.khattignavong@pasteur.la; 3 Administration Department, Institut Pasteur du Laos, Vientiane 01030, Laos; d.phonekeo@pasteur.la; 4 National Center for Laboratory and Epidemiology, Ministry of Health, Vientiane 01030, Laos; bkhamphaphongphane@gmail.com; 5 Department of Communicable Disease Control, Ministry of Health, Vientiane 01030, Laos; phonepadithxangsayarath@gmail.com

**Keywords:** dengue, Lao PDR, serotype, epidemiology, phylogeny

## Abstract

Dengue fever is a widespread mosquito-borne viral disease caused by infections with dengue virus (DENV). Since its initial detection in 1979, the disease has posed a significant public health threat to the Lao People’s Democratic Republic (Lao PDR). Surveillance is crucial for understanding the circulation of DENV in endemic regions and identifying potential hot spots with higher-than-expected case numbers of dengue fever. In this study, we present the results from our surveillance activities in the Lao PDR spanning 2020–2023. While quarantine restrictions from the COVID-19 pandemic posed substantial disruptions to performing DENV surveillance, over 8800 samples were tested during this period, with a positive rate of close to 60%. Cases were reported from all three regions (northern, Central, and southern) of the Lao PDR. Three circulating serotypes (DENV-1, DENV-2, and DENV-4) were detected, with DENV-1 dominant in 2021 and 2022, while DENV-2 was dominant in 2020 and 2023. Phylogenetic analyses suggest that the genotypes of DENV-1, DENV-2, and DENV-4 were closely related to corresponding isolates from neighboring countries. These findings provide an update on the nature of DENV cases detected in the Lao PDR and underscore the critical importance of sustaining a robust surveillance network to track infections.

## 1. Introduction

Dengue is a vector-borne disease of significant global public health concern, with a dramatic rise in the number of cases over the past two decades, increasing from 505,430 cases in 2000 to 5.1 million in 2019 worldwide [1]. Dengue is a viral infection transmitted to humans through the bites of infected female Aedes mosquitoes, which are the principal vectors. Initially confined to tropical and subtropical regions, dengue has expanded its endemic presence to 128 countries in recent years, driven by factors such as climate change, urbanization, international travel, and inadequate mosquito control [2,3,4]. In 2023, the World Health Organization (WHO) recorded the highest number of dengue cases to date, with 6.5 million reported cases and over 7300 dengue-related deaths [1]. The clinical manifestations of dengue vary widely, ranging from a mild flu-like illness known as dengue fever (DF) to more severe forms, including dengue hemorrhagic fever (DHF) and dengue shock syndrome (DSS), with DHF and DSS being potentially life-threatening. Symptoms of DF typically include fever, nausea, vomiting, rash, and body aches. In contrast, DHF and DSS can result in severe bleeding and, for the latter, shock, with a mortality rate reaching 20% if left untreated [5].

The etiological agent, dengue virus (DENV), is a single-stranded, positive-sense RNA virus belonging to the *Orthoflavivirus* genus, *Flaviviridae* family [6]. The DENV genome contains a long open reading frame of 10,700 nucleotides encoding a single polyprotein comprising 10 viral proteins: three structural proteins, capsid (C), pre-membrane (prM), and envelope (E), and seven non-structural proteins (NS1, NS2A, NS2B, NS3, NS4A, NS4B, and NS5) [4]. DENV displays four antigenically distinct serotypes (DENV-1, DENV-2, DENV-3, and DENV-4) that share an approximately 60–65% amino acid sequence similarity [7,8]. The antigenic distance between the four serotypes is sufficient to explain the lack of long-term cross-protective immunity. Each serotype can be further divided into several genotypes. A genotype is defined as a group of DENV isolates that have no more than a 6% nucleotide sequence divergence [9,10]. An increased risk of severe disease has been associated with the co-circulation of multiple DENV serotypes and/or genotypes due to the antibody-dependent enhancement (ADE) phenomenon, as well as the presence of certain specific virus isolates [10,11,12]. These findings highlight the critical importance of establishing and maintaining strong surveillance systems to monitor the circulation of DENV serotypes and the potential emergence of new genotypes in endemic regions.

Dengue-like illness was first documented several centuries ago in South and Southeast Asia, where it occurred sporadically on a large scale [13,14]. The first well-documented outbreak of DHF took place in Manila in 1953/54 and was followed by a larger outbreak in Bangkok in 1958 [15]. Since then, dengue outbreaks have been reported across all countries in South and Southeast Asia, including China, Myanmar, Thailand, Cambodia, and Vietnam [2,14]. Between 2001 and 2010, Southeast Asia experienced a yearly average of 2.9 million dengue infections with 816,000 hospitalizations, resulting in approximately 5900 annual deaths [16]. In 2019, the World Health Organization (WHO) reported a significant increase in dengue cases in Asian countries, such as the Lao People’s Democratic Republic (Lao PDR), Cambodia, Vietnam, the Philippines, Malaysia, and Singapore, compared to the same period in the previous year [17].

In the Lao PDR, the first cases of DHF were recorded in 1979, with the first major outbreak occurring in 1985, resulting in 1759 DHF cases. A subsequent larger outbreak followed in 1987, with 6567 cases reported in Vientiane Capital and 3098 cases across four provinces [18]. Over the years, DENV has become a pathogen of significant public health concern in the Lao PDR, leading to the implementation of national syndromic surveillance since 1998. Under this system, epidemiologists from district hospitals are responsible for collecting and submitting aggregated data on suspected cases to the Department of Health daily. The Department of Health consolidates this data and transmits it to the National Center for Laboratory and Epidemiology (NCLE) weekly [19,20]. The national surveillance system reports clinically suspect dengue cases based on the WHO 2009 classification, which includes dengue with and without warning signs, as well as severe dengue [21]. Notably, laboratory confirmation is not required for reporting in the national framework [22]. This system has enabled the report of suspect dengue cases in the Lao PDR, such as the 2010 outbreak with 22,890 cases and 46 deaths, the 2013 outbreak with 44,171 cases and 95 deaths, and the 2019 outbreak with 39,901 cases and 76 deaths [20,22]. Moreover, all four DENV serotypes have been reported in the Lao PDR, with shifts in the dominant serotype observed during major outbreaks [23,24,25,26,27]. For instance, DENV-3 and DENV-2 were the dominant serotypes during the 2013 and 2019 outbreaks, respectively [20,22].

Quarantine-based public health measures taken during the COVID-19 pandemic caused substantial disruption to the dengue surveillance system in the Lao PDR. As such, there is little information on the situation of DENV infections in the Lao PDR during these years. To address this knowledge gap, we present our dengue surveillance data between 2020 and 2023 to provide a more updated view of the situation of dengue disease in the Lao PDR.

## 2. Materials and Methods

### 2.1. Human Sample Collection

Blood samples (5 mL of venous blood in EDTA tubes) were collected by clinicians from the arbovirus surveillance hospital network in Vientiane Capital and 9 of the Lao provinces as previously described [24,25,26,27,28]. Briefly, suspected patients presenting with dengue fever symptoms matching with the WHO case definition (fever onset ≥38 °C for less than 7 days with at least one of the following accompanying symptoms: headache, myalgia, arthralgia, retro-orbital pain, digestive symptoms, or hemorrhage) were included in this study after obtaining informed consent. Samples were stored at 4 °C before and during transportation to the Institut Pasteur du Laos (IPL), where laboratory diagnosis and molecular analysis were performed. The samples were collected under a national surveillance program, for public health purposes, and, therefore, no ethics committee approval was needed. Nevertheless, all the adult participants provided written informed consent; for children, the consent was given by a parent or a legal guardian to use the samples in the future for research purposes.

### 2.2. Viral RNA Extraction, Dengue Virus Screening, and Serotype Identification

Viral RNA was extracted from plasma samples using purification kits (NucleoSpin RNA virus kit or NucleoSpin 8 virus kit, Macherey-Nagel, Düren, Germany; or Nucleic Acid Extraction Rapid kit, Jiangsu Bioperfectus Technologies, Taizhou, China) following manufacturer instructions. Samples were first screened with pan-DENV real-time RT-PCR (qRT-PCR) [29,30]. Plasma samples that tested negative for DENV by qRT-PCR were screened by the Bioline Dengue Duo NS1 Antigen + Antibodies Combo rapid test (Abbott Diagnostics, Yongin, Republic of Korea) for detection of DENV NS1, anti-DENV IgM, and anti-DENV IgG. The serotype was identified by a multiplex qRT-PCR among the qRT-PCR-DENV-positive samples [30].

### 2.3. Envelope Gene Sequencing

Envelope (E) gene sequencing (1485 nucleotides (nt)) was performed on a panel of DENV strains selected based on their geographical origin, the year of collection, the Ct value for the pan-DENV qRT-PCR, and the availability of the original clinical specimen. Specific amplification was performed using serotype-specific primer sets designed to produce three or four overlapping amplicons, as previously described [25,26,27]. First Stand cDNA was generated using a Maxima H Minus First Stand cDNA Synthesis Kit (Thermo Fischer Scientific, Vilnius, Lithuania), and the PCR was performed using a Phusion Flash High-Fidelity PCR Master Kit (Thermo Fischer Scientific) following manufacturer instructions. The PCR products were purified with ExoSAP-IT PCR Product Cleanup Reagent (Thermo Fisher Scientific) and sequenced using a BigDye Terminator v3.1 Cycle Sequencing Kit (Applied Biosystems, Vilnius, Lithuania) following manufacturer instructions. Sequence chromatograms for both strands were obtained using a 3500xL Genetic Analyzer apparatus (Applied Biosystems).

### 2.4. Phylogenetic Analysis

The raw sequences were analyzed and edited using Unipro UGENE software v46.0 [31]. Multiple-sequence alignments of DENV-1, DENV-2, and DENV-4 sequences were constructed with a subset of related sequences from BLASTn searches and representative genotype sequences collected from GenBank (https://www.ncbi.nlm.nih.gov/nucleotide/, accessed on 4 November 2024). The alignments were generated using the MAFFT program v7.467 [32]. For phylogenetic analysis, maximum likelihood (ML) trees were constructed using the IQ-Tree web server (http://iqtree.cibiv.univie.ac.at/, accessed on 8 November 2024) [33] with automatic substitution model selection using ModelFinder [34] and an ultrafast bootstrap of 1000 replications [35]. The trees were subsequently edited using FigTree v1.4.4 software (http://tree.bio.ed.ac.uk/software/, accessed on 19 September 2023). The nucleotide sequences of the complete E gene obtained in this study were submitted to GenBank, and their accession numbers (PQ775559 to PQ775659) are listed in Table S1.

### 2.5. Data Analysis

Demographic data were extracted from the de-identified database of the surveillance network. The data were analyzed with GraphPad Prism 9.0 software. The unpaired non-parametric Mann–Whitney test was used to compare the percentages of confirmed DENV cases in females and males. Two samples with missing gender information were excluded for gender analysis. The non-parametric Kruskal–Wallis test, followed by Dunn’s correction, was used to compare the percentages of confirmed DENV cases among age groups and provinces. For analysis by age groups and provinces, 39 and 17 patients with missing data were excluded from the respective analyses.

## 3. Results

### 3.1. Dengue Surveillance Results Between 2020 and 2023

**Figure 1:**
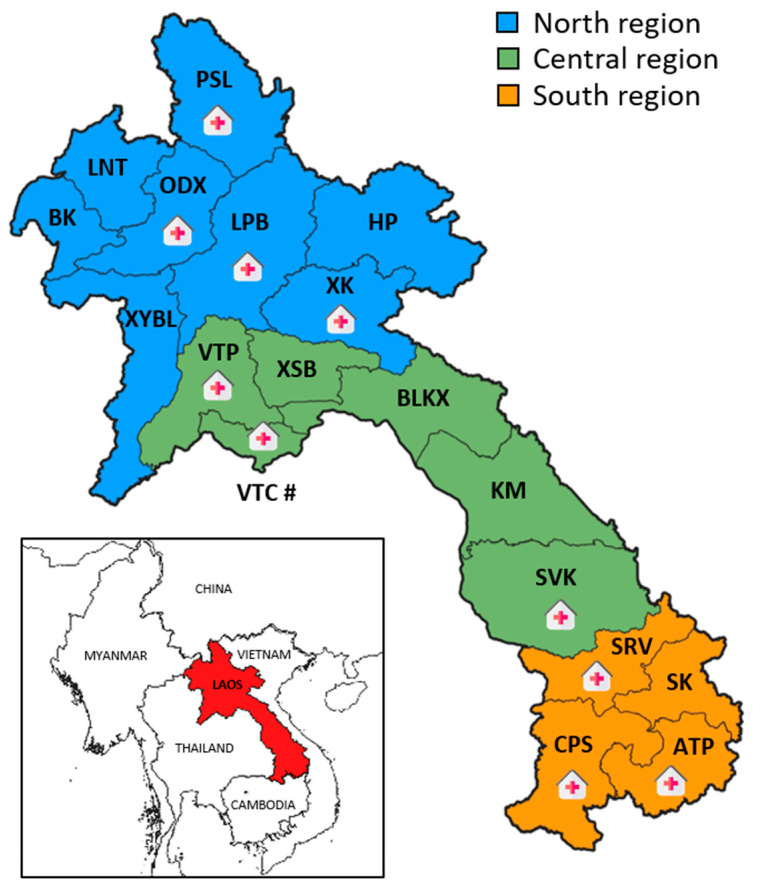
Map of the Lao PDR with sentinel sites. The provinces of the Lao PDR are grouped in one of three geographical regions: the North region (in blue), including Bokeo (BK), Huaphan (HP), Luangnamtha (LNT), Luangprabang (LBP), Phongsaly (PSL), Oudomxay (ODX), Xiengkouang (XK), and Xayabouly (XYBL); the Central region (in green), including Bolikhamxay (BLKX), Khammouane (KM), Savannakhet (SVK), Vientiane Capital (VTC), Vientiane Province (VTP), and Xaysomboune (XSB); and the South region (in orange), including Attapeu (ATP), Champasack (CPS), Saravane (SRV), and Sekong (SK). Sentinel sites are marked with hospital icons, with 13 sites specifically located within Vientiane Capital, indicated by a # symbol.

For this study, the dengue surveillance network included 22 sentinel sites across Vientiane Capital and nine provinces in the Lao PDR: Attapeu, Champasack, Luangprabang, Oudomxay, Phongsaly, Savannakhet, Saravane, Vientiane Province, and Xiengkhouang. These sentinel sites were strategically distributed across the North, Central, and South regions of the Lao PDR (Figure 1).

**Figure 2:**
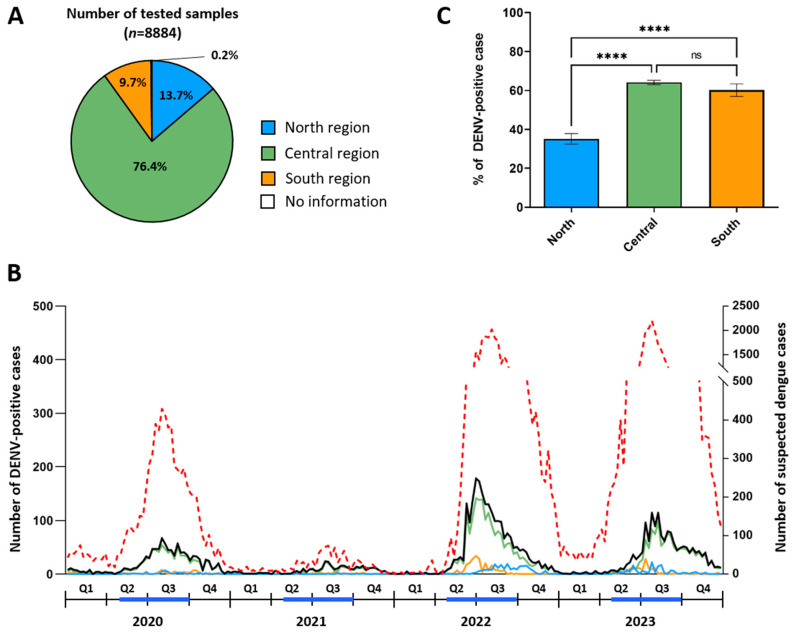
Distribution of samples tested and DENV-positive cases according to geographical origin and time. (**A**) Number of tested samples according to geographical origin. (**B**) Weekly record of the number of DENV-positive cases and national suspected dengue cases between 2020 and 2023. The black line represents the DENV-positive cases detected in this study, and the dotted red line represents the number of suspected dengue cases reported at the national level by the National Center for Laboratory and Epidemiology (NCLE) in the Lao PDR (*n* = 77,484). The blue line in the timeline represents the rainy season period (from May to September). (**C**) Percentage of DENV-positive cases according to geographical origin. The error bars indicate 95% confidence intervals. The *p*-values are represented as follows: ns = non-significant, **** = <0.0001. In the three panels, the same color code as that defined in Figure 1 is used to represent the origin of the samples and/or the DENV-positive cases (blue = North, green = Central, orange = South).

From 2020 to 2023, a total of 8884 samples were collected through our arbovirus surveillance system from suspected dengue patients originating from all provinces of the Lao PDR. The majority of the samples, 6784 (76.4%), were obtained from patients from the Central provinces, including 6093 (68.6%) from patients from Vientiane Capital, where 13 of the 22 sentinel sites were located. Additionally, 1219 (13.7%) blood samples were collected from patients from northern provinces and 864 (9.7%) from southern provinces (Figure 2A). Seventeen patients (0.2%) had no recorded province of origin. The gender ratio of the dengue-suspected patients was approximately 1.01 females (*n* = 4467) to 1 male (*n* = 4415), with gender data missing for 2 patients (Table 1). The mean age of the patients was 28.09 years, ranging from 1 month to 100 years old, with age data missing for 39 patients.

Of the 8884 samples tested, 5308 (59.8%) were DENV-positive through real-time DENV qRT-PCR and/or DENV NS1 detection, ranging from 50.1% in 2021 to 63.4% in 2023 (Table 1). The gender ratio of the DENV-positive cases was approximately 1.09 females (*n* = 2763) to 1 male (*n* = 2545), with the percentage of DENV-positive cases slightly higher in females than in males (61.9% (95% confidence interval (CI): 60.4–63.3) vs. 57.6% (95% CI: 56.2–59.1) (*p* < 0.0001)) (Table 1). The percentage of DENV-positive cases varied significantly across age groups. Among children aged 6 to 10 years, 62.1% (95% CI: 58.7–65.5) were confirmed as DENV-positive cases, a rate significantly higher (*p*-value <0.0001) than that observed in children under 5 years old, which was 42.5% (95% CI: 38.6–46.4). The highest percentage of DENV-positive cases was observed in adolescents and young adults, with 69.8% (95% CI: 67.6–71.9) in the 11–20 age group and 67.5% (95% CI: 65.6–69.4) in the 21–30 age group. Among middle-aged adults (31 to 50 years), the rate of DENV-positive cases declined, with 58.3% (95% CI: 55.8–60.9) in those aged 31 to 40 and 52.7% (95% CI: 49.4–56.5) in those aged 41 to 50. This downward trend continued among older adults, reaching the lowest level of DENV-positive cases at 25.9% (95% CI: 19.1–32.7) in those aged over 71 years (Table 1).

Between 2020 and 2023, the number of DENV-positive cases peaked between June and November, aligning with the disease incidence observed by the national dengue surveillance in the Lao PDR and coinciding with and following the rainy season (Figure 2B). In 2022, the peak of DENV-positive cases in the northern region appeared slightly delayed, beginning in mid-July, while the southern region experienced a more restricted peak period from June to August. In contrast, 2023 saw a prolonged peak in the Central region, lasting from June through November, while the northern and southern regions had a more concentrated peak period, with DENV-positive cases primarily occurring between June and August (Figure S1).

**Figure 3:**
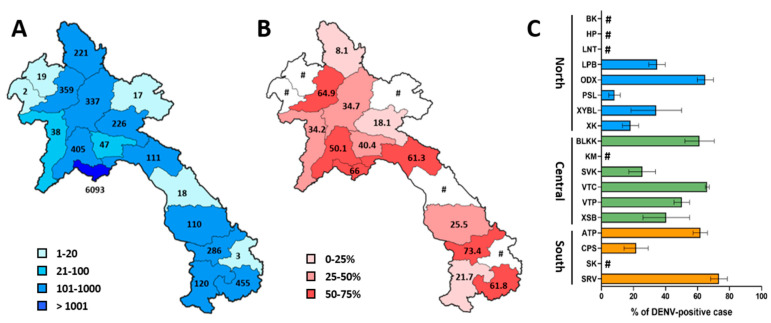
Geographical distribution of tested samples and DENV-positive cases in the Lao PDR between 2020 and 2023. (**A**) The blue-scale map shows the total number of samples tested in this study by province, with the total for each province indicated in black. (**B**) The red-scale map indicates the percentage of DENV-positive cases per province, with positivity rates shown in black. Provinces with fewer than 20 tested samples are represented in white and marked with a #. (**C**) The graph displays the percentage of DENV-positive cases per province, colored by region: blue for the North, green for the Central, and orange for the South regions. Province letter codes follow those previously defined in the legend of Figure 1. Provinces with fewer than 20 tested samples are marked with a #. The error bars indicate 95% confidence intervals.

Overall, the percentage of DENV-positive cases was lower in the northern region at 35.1%, compared to the Central and southern regions, which had detection rates of 64.1% and 60.2%, respectively (Figure 2C). This trend, with a lower percentage of DENV-positive cases in the northern region, was consistently observed each year from 2020 to 2023 (Figure S2). Notable differences existed between provinces within these three regions. To reduce the bias of interpretation, the percentage of DENV-positive cases was calculated only for provinces in which over 20 samples were collected (Figure 3A). In the northern region, the rate of DENV-positive cases ranged from 8.1% in Phongsaly to 64.9% in Oudomxay (Figure 3B,C). In the Central region, Savannakhet had the lowest percentage of DENV-positive cases at 25.5%, while the highest percentages in this region were recorded in Bolikhamxay and Vientiane Capital, with 61.3% and 66%, respectively (Figure 3B,C). In the southern region, Champasack had a lower percentage of DENV-positive cases, at 21.7%, compared to Saravane, which recorded a high rate of DENV-positive cases, at 73.4% (Figure 3B,C).

A similar trend was observed when data from 2020 to 2023 were analyzed annually, focusing on provinces that collected over 10 samples in a particular year (Figure S3). In the northern region, the lowest percentages of DENV-positive cases were recorded in Phongsaly (between 7.2% and 9.8%), and the highest ones were observed in Oudomxay (between 60.5 and 68.1%) (Figure S2). In the Central region, Vientiane Capital had DENV-positive cases consistently exceeding 50% over the four years, and Savannakhet showed the lowest rates of DENV-positive cases in 2022 (18.2%) and 2023 (41%) (Figure S2). In the southern region, Champasack had the lowest percentages of DENV-positive cases in 2022 (18.6%) and 2023 (26.1%). In contrast, Saravane exhibited the highest DENV-positive case rates during 2020–2023 (between 60.9 and 81.9%) (Figure S2). Across all provinces, 2021 recorded the lowest percentages of confirmed DENV cases, with only Vientiane Capital and Saravane showing positive rates above 50% (Figure S2).

### 3.2. Serotype Circulation in Lao PDR Between 2020 and 2023

**Figure 4:**
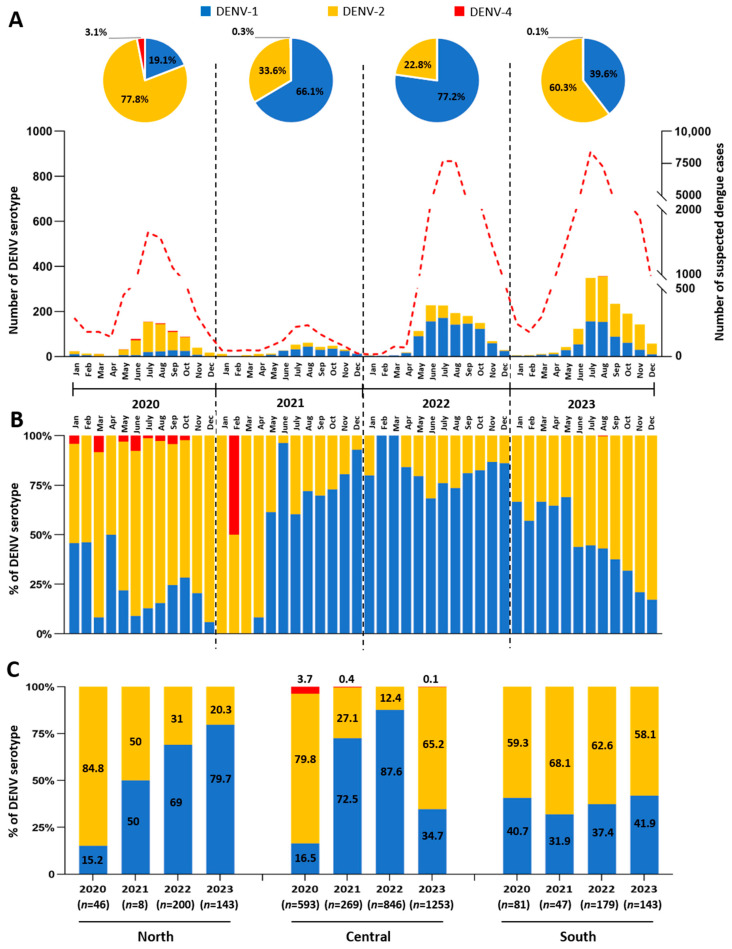
Distribution of DENV serotypes in the Lao PDR from 2020 to 2023. (**A**) The graph shows the number of each serotype detected in this study by month, and each serotype is represented by a different color: blue (DENV-1), yellow (DENV-2), and red (DENV-4). The dotted red line denotes the total number of suspected dengue cases at the national level, while the histogram indicates the number of viruses for which the serotype was identified. Yearly pie charts display the percentage breakdown of each serotype. (**B**) The graph shows the percentage of each DENV serotype by month using the same color code for serotypes as in (**A**). (**C**) The three panels illustrate the percentage of each DENV serotype by region (North, Central, South) and year, using the same color code to identify the serotypes as in panel (**A**).

Among the dengue-positive cases (*n* = 5308), 4668 cases (88%) were identified as DENV-positive by qRT-PCR, while 640 cases (12%) cases were confirmed via NS1 antigen detection. DENV serotypes were determined by real-time qRT-PCR for 3810 cases, representing 4.9% of the national suspected dengue cases overall. By individual years, the representativeness of the samples in this study to the overall national suspected clinical case numbers is as follows: 8.8% in 2020, 20.8% in 2021, 3.8% in 2022, and 4.4% in 2023 (Figure 4A).

Between 2020 and 2023, DENV-1 and DENV-2 were the dominant serotypes identified, accounting for 50.1% and 49.3% of DENV-positive cases, respectively. Co-infections with DENV-1 and DENV-2 were detected in four cases in 2023. Only 24 cases of DENV-4 (0.6%) were detected (22 cases in 2020, 1 case in 2021, and 1 case in 2023), while no cases of DENV-3 were identified. However, the proportions of DENV-1 and DENV-2 varied throughout the four years, showing distinct circulation dynamics. In 2020, DENV-2 was the dominant serotype, representing 77.8% of viruses serotyped in this year. The proportion of DENV-2 increased from 50% of cases in January 2020 to 94% by December 2020. DENV-2 remained the most frequent serotype until April 2021. In May 2021, however, a shift occurred, with DENV-1 becoming the dominant serotype, representing 61.5% of all circulating serotypes that month. As a result, DENV-1 became and remained the major serotype throughout 2021 and 2022, accounting for 66.1% and 77.2% of positive samples, respectively. This trend persisted until May 2023, when DENV-2 once again became the dominant serotype, representing 82.8% of all positive DENVs detected by December 2023 (Figure 4A,B).

Geographically, DENV-1 and DENV-2 displayed distinct circulation patterns over the four-year period. In the northern region, DENV-1 circulation substantially increased, from 15.2% in 2020 to 79.7% in 2023 (Figure 4C). Serotype trends in the Central region mirrored the national pattern, with DENV-2 as the major serotype in 2020 (79.8%) and again in 2023 (65.2%), while DENV-1 was dominant in 2021 (72.5%) and 2022 (87.6%) (Figure 4C). DENV-4 was only detected in the Central region, specifically in Vientiane Capital, in 3.7% of DENV-positive cases in 2020 and one case in 2021 and 2023. In contrast, DENV-2 consistently dominated in the South region, comprising between 58.1% and 68.1% of positive samples during the 2020–2023 period (Figure 4C).

### 3.3. DENV Sequence Analysis

A panel of 101 sequences, including 42 DENV-1, 40 DENV-2, and 19 DENV-4, were selected for sequencing and phylogenetic analysis. These sequences were selected to represent a range of collection times and locations (Table S1). Sequence analyses focused on the complete coding sequence of the E gene (1485 nt) to investigate the circulation dynamics of these three DENV serotypes in the Lao PDR from 2020 to 2023.

**Figure 5:**
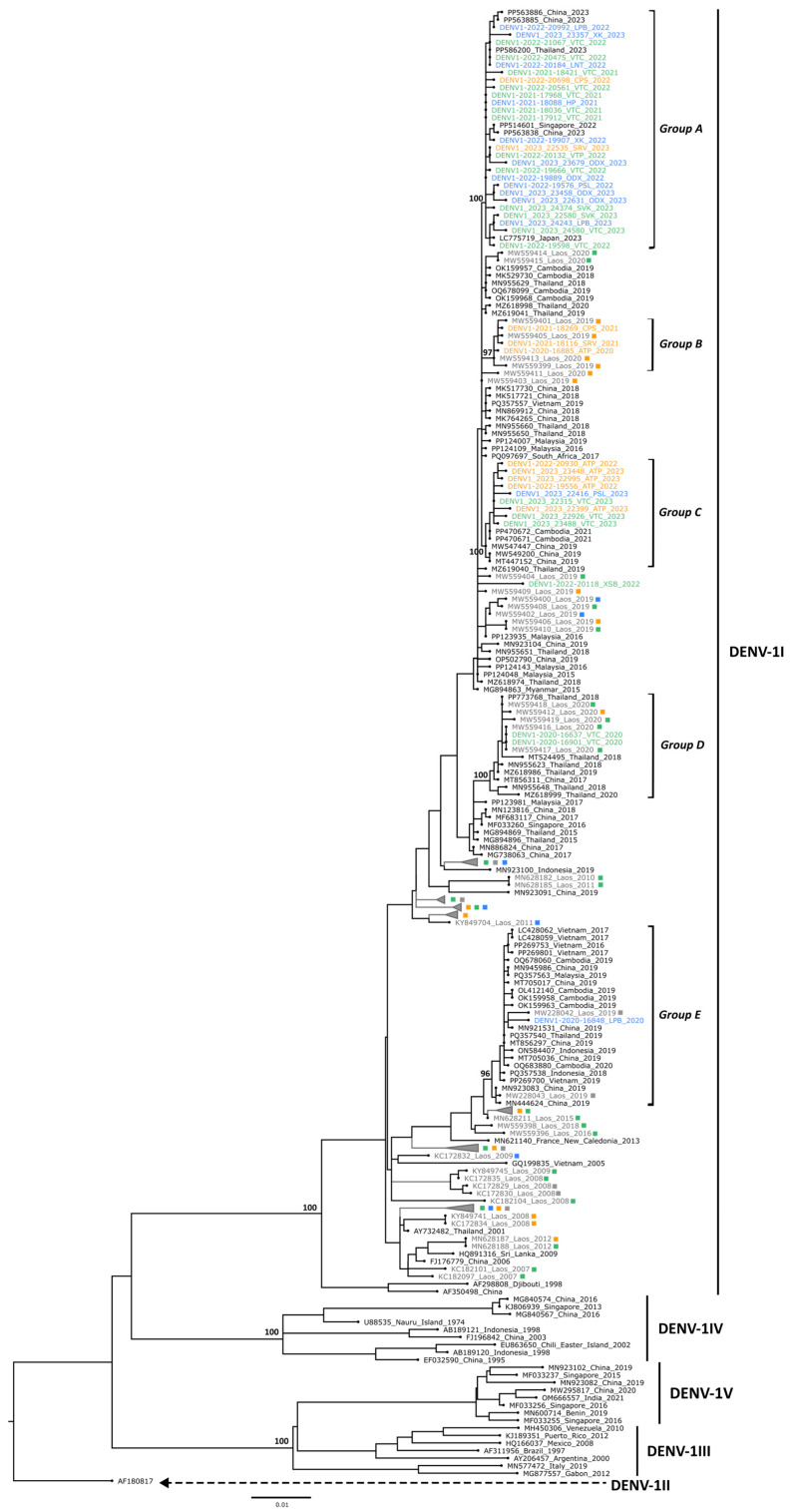
Maximum likelihood tree of the DENV-1 envelope sequence. A total of 42 new DENV-1 sequences were combined with 280 DENV-1 sequences selected from GenBank, including 172 obtained from Lao strains. The nucleotide sequences were aligned by MAFFT, and a phylogenetic tree was constructed using IQ-Tree using the TIM2 + F + I + G4 model with 1000 ultrafast bootstrap replicates. The major genotypes are labelled according to a previously published study [14]. Bootstrap values for major genotypes and groups (groups A–E) containing sequences generated in this work are displayed at the tree nodes. The sequences generated in this work are color-coded by region: blue (North), green (Central), and orange (South), while previously reported Lao sequences are in grey. The squares indicate the geographical origin of the previously reported Lao sequences: blue (North), green (Central), orange (South), and grey (unspecified origin).

**Figure 6:**
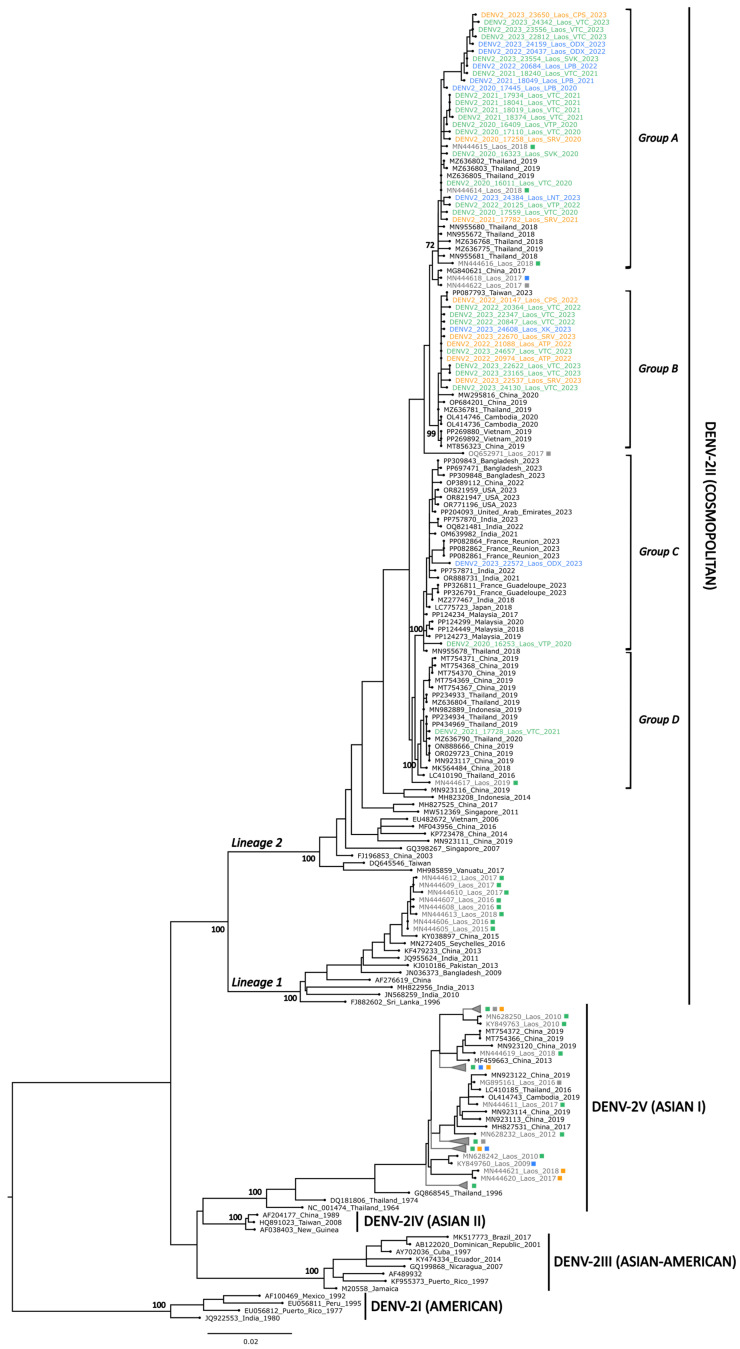
Maximum likelihood tree of the DENV-2 envelope sequence. A total of 40 new DENV-2 sequences were combined with 170 DENV-2 sequences selected from GenBank, including 61 obtained from Lao strains. The nucleotide sequences were aligned by MAFFT, and a phylogenetic tree was constructed using IQ-Tree using the TIM2 + F + I + G4 model with 1000 ultrafast bootstrap replicates. The major genotypes and lineages are labelled according to a previously published study [14]. Bootstrap values for major genotypes, lineages, and groups (groups A–D) containing sequences generated in this work are displayed at the tree nodes. The sequences generated in this work are color-coded by region: blue (North), green (Central), and orange (South), while previously reported Lao sequences are in grey. The squares indicate the geographical origin of the previously reported Lao sequences: blue (North), green (Central), orange (South), and grey (unspecified origin).

**Figure 7:**
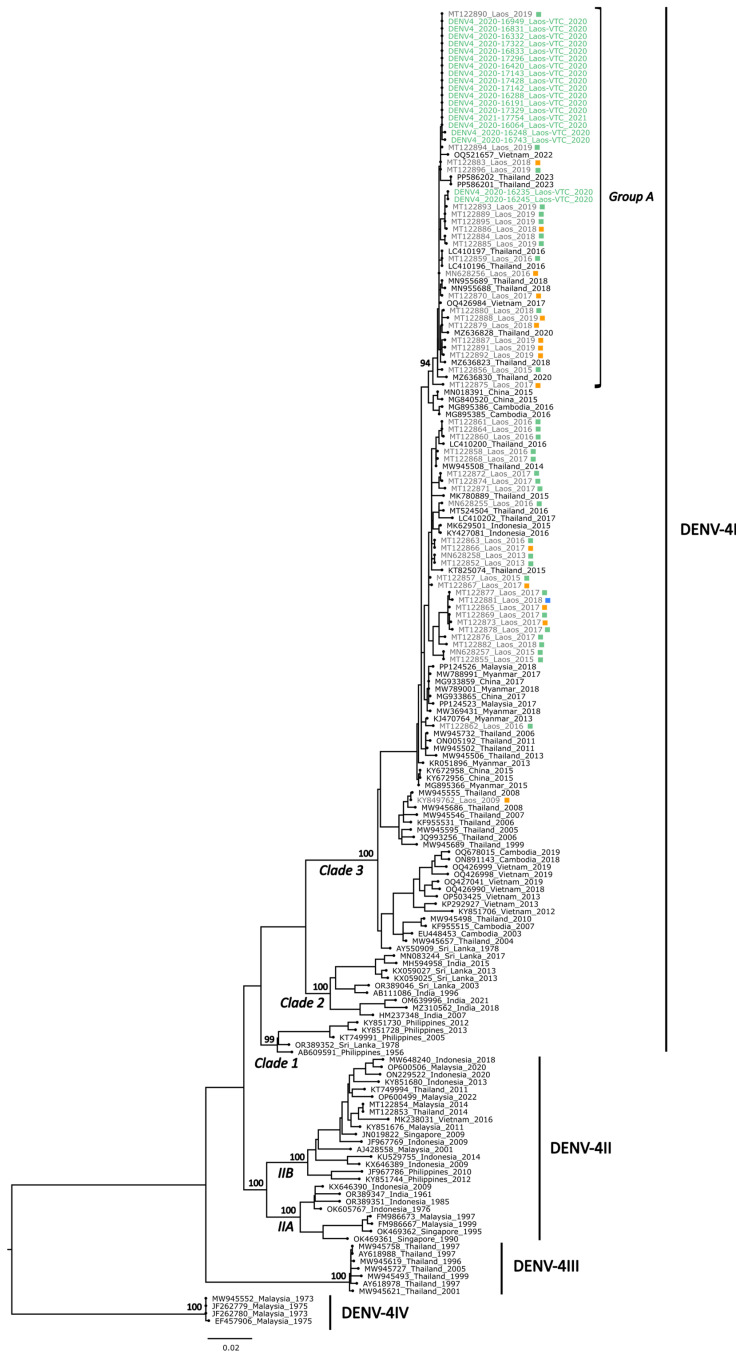
Maximum likelihood tree of the DENV-4 envelope sequence. A total of 19 new DENV-4 sequences were combined with 158 DENV-4 isolate sequences selected from GenBank, including 48 obtained from Lao strains. The nucleotide sequences were aligned by MAFFT, and a phylogenetic tree was constructed using IQ-Tree using the GTR + F + G4 model with 1000 ultrafast bootstrap replicates. The major genotypes, lineages, and clades are labelled according to a previously published study [14]. Bootstrap values for the major genotypes, lineages, clades, and groups, including sequences generated in this work, are displayed at the tree nodes. The sequences generated in this work are color-coded by region: blue (North), green (Central), and orange (South), while previously reported Lao sequences are in grey. The squares indicate the geographical origin of the previously reported Lao sequences: blue (North), green (Central), and orange (South).

The sequences generated were aligned with previously published DENV sequences from the Lao PDR [23,24,25,26,27] and DENV sequences from neighboring countries obtained from GenBank to inform the DENV molecular epidemiology in the Lao PDR and Southeast Asia. Phylogenetic trees indicated that the sequences generated belonged each time to a unique genotype: genotype I for DENV-1 (DENV-1I), genotype II for DENV-2 (DENV-2II), and genotype I for DENV-4 (DENV-4I) (Figure 5, Figure 6 and Figure 7).

#### 3.3.1. DENV-1 Phylogeny

A total of 42 DENV-2 sequences from strains collected between 2020 and 2023 from 13 Lao provinces (Attapeu, Champasack, Huaphanh, Luangnamtha, Luangprabang, Oudomxay, Phongsaly, Saravane, Savannakhet, Vientiane Capital, Vientiane Province, Xaysomboune, and Xiengkhouang) were generated. The strains sequenced were representative of the three main regions of the Lao PDR (North (*n* = 13), Central (*n* = 19), and South (*n* = 10)). The phylogenetic analysis indicated that the 42 E gene sequences belonged to DENV-1 genotype I (DENV-1I) (Figure 5).

The E gene sequences of DENV-1 strains detected from 2020 to 2023 displayed a nucleotide identity ranging from 95.9% to 100% (99.1% to 100% at amino acid level) and were distributed across five distinct groups (A to E). The largest group, group A, comprised 26 new DENV-1 sequences that shared over 99.3% of nucleotide identity between each other and clustered with sequences of strains from China, Japan, Singapore, and Thailand collected in 2022 and 2023 (>99.3% of nucleotide identity, bootstrap value of 100). Group B, which appeared to be specific to the southern region of the Lao PDR, contained three recent DENV-1 sequences and four previously generated from Lao strains collected in 2019 and 2020. These seven sequences included in group B displayed a high overall nucleotide identity (>99.7% of identity at the nucleotide level, bootstrap value of 97). Group C was made of nine new DENV-1 sequences that shared an overall nucleotide identity above 99.5%. The sequences of this group clustered with sequences of strains originating from Cambodia and China collected in 2019 and 2021 (>99.5% of nucleotide identity, bootstrap value of 100). The sequences of two Lao strains collected in Vientiane Capital in 2020 were assigned to group D, which included sequences from China, the Lao PDR, and Thailand collected between 2017 and 2020 (>99.1% nucleotide identity, bootstrap value of 100). The E gene sequences of these two Lao strains were 100% identical to each other and to two previously described Lao strains identified in Vientiane in 2020 (GenBank accession numbers: MW559416 and MW559417) [27]. The sequence of the DENV-1 strain number 2022-20118, collected in 2022 in Xaysomboune, shared 99.3% of nucleotide identity with the sequence of a strain found in 2015 in Malaysia (PP124048) that remained unrelated to any defined group. The most divergent sequence, obtained from strain 2020-16848, collected in 2020 in Luangprabang, shared between 95.9% and 96.6% identity at the nucleotide level and 99.1% and 99.7% identity at the amino acid level with the other Lao DENV-1 strain sequences described in this study. This strain clustered within group E, which included sequences of strains from Cambodia, China, Indonesia, the Lao PDR, Thailand, and Vietnam, collected between 2016 and 2020 (>99.3% of shared nucleotide identity, bootstrap value of 100).

#### 3.3.2. DENV-2 Phylogeny

A total of 40 DENV-2 isolates collected between 2020 and 2023 from 10 Lao provinces (Attapeu, Champasack, Luangnamtha, Luangprabang, Oudomxay, Saravane, Savannakhet, Vientiane Capital, Vientiane Province, and Xiengkhouang) were successfully sequenced. These samples originated from the three main regions of the Lao PDR (North (*n* = 8), Central (*n* = 24), South (*n* = 8)). Phylogenetic analysis demonstrated that all these 40 E gene sequences belonged to genotype II (DENV-2II), also known as the Cosmopolitan genotype, and grouped in lineage 2, also referred to as lineage B (Figure 6) [14].

Overall, the sequences of the DENV-2 strains detected between 2020 and 2023 displayed a high nucleotide identity, ranging from 97.4% to 100% (98.9 to 100% of identity at amino acid level) and were distributed across four distinct groups (groups A–D). The major group, namely group A, comprised 24 sequences of recent DENV-2 strains, sharing over 98.8% of nucleotide identity. The sequences of these strains grouped with the sequences of three Lao strains detected in 2018 and nine Thai strains collected in 2018 and 2019 (>98.7% of shared nucleotide identity, bootstrap value of 72). Group B included 13 new DENV-2 sequences, with nucleotide identity above 99.6%, which clustered with sequences from Cambodia, China, Vietnam, and Taiwan collected between 2019 and 2023 (>99.4% of shared nucleotide identity, bootstrap value of 99). In group D, a single sequence of a Lao strain from 2021 (collected in Vientiane Capital) clustered with sequences from China, Indonesia, and Thailand collected between 2016 and 2020 (>99.5% nucleotide identity, bootstrap value of 100). Interestingly, the sequences of two Lao DENV-2 strains from 2020 and 2023 clustered in group C, which also included sequences from a wide geographical range of North America (Florida, USA); the Caribbean (Guadeloupe); East Africa (Reunion); West Asia (United Arab Emirates); South Asia (Bangladesh, India and Sri Lanka); Southeast Asia (Malaysia and Thailand); and East Asia (Japan) (>99.1% nucleotide identity, bootstrap value of 100).

#### 3.3.3. DENV-4 Phylogeny

A total of 19 DENV-4 viruses collected in 2020 (*n* = 18) and 2021 (*n* = 1) from Vientiane Capital were successfully sequenced. Phylogenetic analysis revealed that these 19 E gene sequences belonged to clade 3 of DENV-4 genotype I (DENV-4I) (Figure 7) [14].

The sequences of these DENV-4 strains exhibited a high nucleotide identity of over 99.3% (99.4% of amino acid identity), with 15 sequences showing 100% identity. These 19 sequences formed a distinct group (group A), which included sequences from the Lao PDR (collected between 2015 and 2019, from the Central and South regions), Thailand, and Vietnam. The nucleotide sequence identity ranged from 99 to 100%, with a bootstrap support of 94. Additionally, the sequences of these 2020–2021 strains displayed a maximum nucleotide divergence of 3.8% (amino acid divergence of 1.7%) with previously published DENV-4I Lao sequences, with the highest nucleotide divergence observed with the sequence of the first DENV-4 strain identified in the Lao PDR (KY849762) and collected in 2009 in Saravane.

## 4. Discussion

The 8884 cases tested in this study represent 11.4% of the 77,484 total suspected dengue cases reported by the national dengue surveillance program during the 2020–2023 period. The proportion of cases tested in the study relative to the total number of suspected cases ranged from 7.1% in 2023 to 46.9% in 2021. The implementation of public health measures during the COVID-19 pandemic may have contributed to these variations. Indeed, quarantine-based measures limited the ability of patients to seek medical care and led to logistical disruptions in sample transportation to laboratories in some provinces. In addition, new policies resulted in the diversion of limited resources in the Lao PDR towards combating COVID-19, inadvertently disrupting surveillance programs for other diseases, such as dengue [36,37,38]. Additionally, the overlap in early symptoms between COVID-19 and dengue, such as fever, muscle pain, and fatigue, may have led to challenges in diagnostic testing and potential misclassification of some cases [39,40]. As a consequence, while the data presented here showed some trends, it was not possible to perform a more robust epidemiological analysis since the clinical samples sent to the IPL for virological confirmation were not selected to be representative of the total number of dengue cases reported at the national level.

However, the sequences generated from this work allowed us to explore the spatial and temporal dynamics of DENV circulation in the Lao PDR and in neighboring countries. These data are crucial to monitor serotype and genotype shifts to anticipate potential dengue outbreaks. Indeed, the risk of severe dengue increases with the co-circulation of different serotypes and genotypes, a phenomenon often exacerbated by ADE [4,11,12] Moreover, certain DENV isolates may exhibit higher virulence and result in more severe outbreaks [10]. From phylogenetic analyses conducted on selected DENV-1, DENV-2, and DENV-4 nucleotide sequences, the trees showed that these sequences were closely related to sequences of viruses identified in Southeast Asia, specifically belonging to the genotypes DENV-1I, DENV-2II, and DENV-4I, respectively.

The DENV-1 sequences belong to genotype DENV-1I, which is the main DENV-1 genotype circulating in Southeast Asia since the 1980s, seemed to originate from Thailand and then spread regionally to Cambodia, Vietnam, Myanmar, and the Lao PDR [14]. The DENV-1 sequences are grouped into five clusters (groups A–E), in which two (groups A and C) represent newly identified clusters. Moreover, group B showed geographic specificity, consisting solely of strains from the southern provinces. These findings suggest ongoing DENV-1 evolution and multiple introductions, likely from neighboring countries such as Thailand, China, or Cambodia, which could form local clusters that circulate briefly before replacement, as described previously [14,24,27]. The newly sequenced DENV-2 strains belong to the DENV-2II genotype, also known as the Cosmopolitan genotype, which also emerged in recent years in neighboring countries, such as Vietnam and Cambodia [14]. The DENV-2 sequences clustered into four groups (groups A–D). Group A contains 24 new Lao strains, with Lao and Thai strains from 2018–2019, suggesting their wide circulation in both countries since then. Group B, containing 13 new Lao strains only from 2022 and 2023, also contains strains from Cambodia, China, Vietnam, and Taiwan, collected in 2019 and 2021, suggesting a new introduction in the Lao PDR, possibly in 2022. Group C corresponds to a global cluster, reflecting the broad dissemination of the Cosmopolitan genotype and invasive potential through international transmission routes [41,42,43]. To date, only two DENV-4 genotype II (DENV-4II) strains have been detected in the Lao PDR, both from European tourists traveling in Southeast Asia in 2014 who subsequently came down with dengue in Vientiane Capital [26]. In contrast, since 2009, all of the autochthonous Lao DENV-4 strains, mostly detected in the Central and southern regions, belong to the DENV-4I genotype, which has been the dominant DENV-4 genotype for decades in mainland Southeast Asia, particularly in Cambodia, Thailand, and Vietnam [14], and formed several distinct clusters. Group A, which includes the new DENV-4 sequences from this study, clustered with sequences from the Lao PDR, Thailand, and Vietnam collected from 2016 to 2023, suggesting a wide geographical circulation of this cluster among the neighboring countries, as described previously [14,23,24,25,26].

Surveillance of serotype circulation is important, as differences in DENV serotype patterns can affect the disease dynamics by influencing transmission, the population’s immunity, and disease severity. Indeed, previous studies have suggested that the four DENV serotypes may exhibit varying levels of virulence [9,44,45,46]. Moreover, prospective cohort studies in Asia and Latin America have identified secondary heterotypic DENV infection as an epidemiological risk factor for severe disease [11,47,48,49]. More in-depth seroprevalence studies are needed in the Lao PDR to decipher the immunity status of the local population against the four DENV serotypes, and to understand the impact of host immunity on disease susceptibility.

Between 2020 and 2023, DENV-1 and DENV-2 were the dominant serotypes, accounting for 99.4% of circulating serotypes. Similar findings were reported in Cambodia and Vietnam between 2020 and 2022, with a low detection of DENV-3 and DENV-4 [50,51,52,53,54]. Moreover, our data suggest that the most common DENV serotype detected in the Lao PDR varied by year (Figure 4A). Serotype shifts in the Lao PDR have been previously observed and coincided with major dengue outbreaks, such as in 2013, when DENV-3 emerged, but have not been detected since 2016 [20,24,28]. DENV-1 has circulated in the Lao PDR since 2007, becoming the primary serotype between 2007 and 2011 and then again in 2015, while DENV-2, although less common in the early 2010s, caused a significant outbreak in 2019 [20,23,24,25,27]. Additionally, the data suggest geographical differences in the circulation of DENV in the Lao PDR (Figure 4C). This aligns with a previous study showing that DENV-1 was dominant in Vientiane Capital (Central) and Saravane (South) between 2008 and 2010, but DENV-2 was more common in Luangnamtha (North) between 2008 and 2009 [23]. Similar patterns with serotype shifts over the years coinciding with major outbreaks have been previously reported in Southeast Asia, such as Cambodia. The 2007 outbreak was driven by DENV-3, whereas the 2012 outbreak was driven by DENV-1 and mostly due to a genotype replacement of genotype DENV-1IV by DENV-1I. In contrast, a co-circulation of DENV-1 and DENV-2 was detected during the 2019 outbreak [51].

Variations in the percentages of DENV serotypes over time and location, regional co-circulation of DENV genotypes, and their possible introduction into the Lao PDR from neighboring countries underscore a critical need from a public health viewpoint to perform continuous surveillance to anticipate potential outbreaks. This will help authorities to enact effective, proactive measures (i.e., targeted vector control, improved diagnostic capacity, and public awareness campaigns) to reduce dengue morbidity and mortality. From a research viewpoint, it is important to characterize the antigenic evolution of DENV strains in the region [55,56,57] and the immune status of the local human population against different antigenic variants of DENV to understand the mechanisms behind DENV serotype switches [58,59]. Beyond the Lao PDR, coordinated regional surveillance efforts that integrate virological, immunological, and entomological data against the backdrop of environmental change should be pursued to understand and predict whether the introduction of new DENV genotypes to a new location may result in severe disease and outbreaks for the local populace.

## Figures and Tables

**Table 1:** Demographic characteristics of the 8884 suspected dengue patients in the Lao PDR between 2020 and 2023.

	Number of Samples Tested	Number of DENV- Positive Cases #	% of DENV- Positive Cases	95% CI *
**Year**				
2020	1849	1042	56.4%	54.1–58.6
2021	730	366	50.1%	46.5–53.8
2022	3778	2298	60.8%	59.3–62.4
2023	2527	1602	63.4%	61.5–65.3
**Gender**				
Female	4467	2763	61.9%	60.4–63.3
Male	4415	2545	57.6%	56.2–59.1
Unknown	2	0	-	-
**Age**				
0–5	624	265	42.5%	38.6–46.4
6–10	778	483	62.1%	58.7–65.5
11–20	1784	1277	69.8%	67.6–71.9
21–30	2411	1628	67.5%	65.6–69.4
31–40	1406	820	58.3%	55.8–60.9
41–50	774	408	52.7%	49.4–56.5
51–60	559	236	42.2%	38.1–46.3
61–70	347	133	38.3%	33.2–43.5
>71	162	42	25.9%	19.1–32.7
Unknown	39	16	41%	24.9–57.2
**Total**	**8884**	**5308**	**59.8%**	**58.7–60.8**

#: DENV-positive cases correspond to samples tested positive by DENV qRT-PCR and/or DENV NS1; *: 95% CI: 95% confidence interval (CI).

## Data Availability

The nucleotide sequences of the complete E gene obtained in this study are available in GenBank (PQ775559 to PQ775659).

## Supplementary Materials

The following supporting information can be downloaded at https://www.mdpi.com/article/10.3390/microorganisms13020318/s1, Table S1: List of the Lao DENV strains selected for sequencing and phylogenetic analysis. Figure S1: Number of DENV-positive cases reported monthly between 2020 and 2023. Figure S2. Percentage of DENV-positive cases according to geographical origin in years 2020–2023. Figure S3. Geographical distribution of samples tested and DENV-positive cases in Lao PDR per year between 2020 and 2023.
