# Supplementary material for: Molecular Epidemiology of Dengue Viruses in Lao People’s Democratic Republic, 2020–2023
Source: Microorganisms. 2025 Feb 1;13(2):318. doi: 10.3390/microorganisms13020318 (PMC11857872; doi:10.3390/microorganisms13020318)
Supplement: Supplementary file 1 [file microorganisms-13-00318-s001.zip › TROUPIN-FigureS1.pdf]

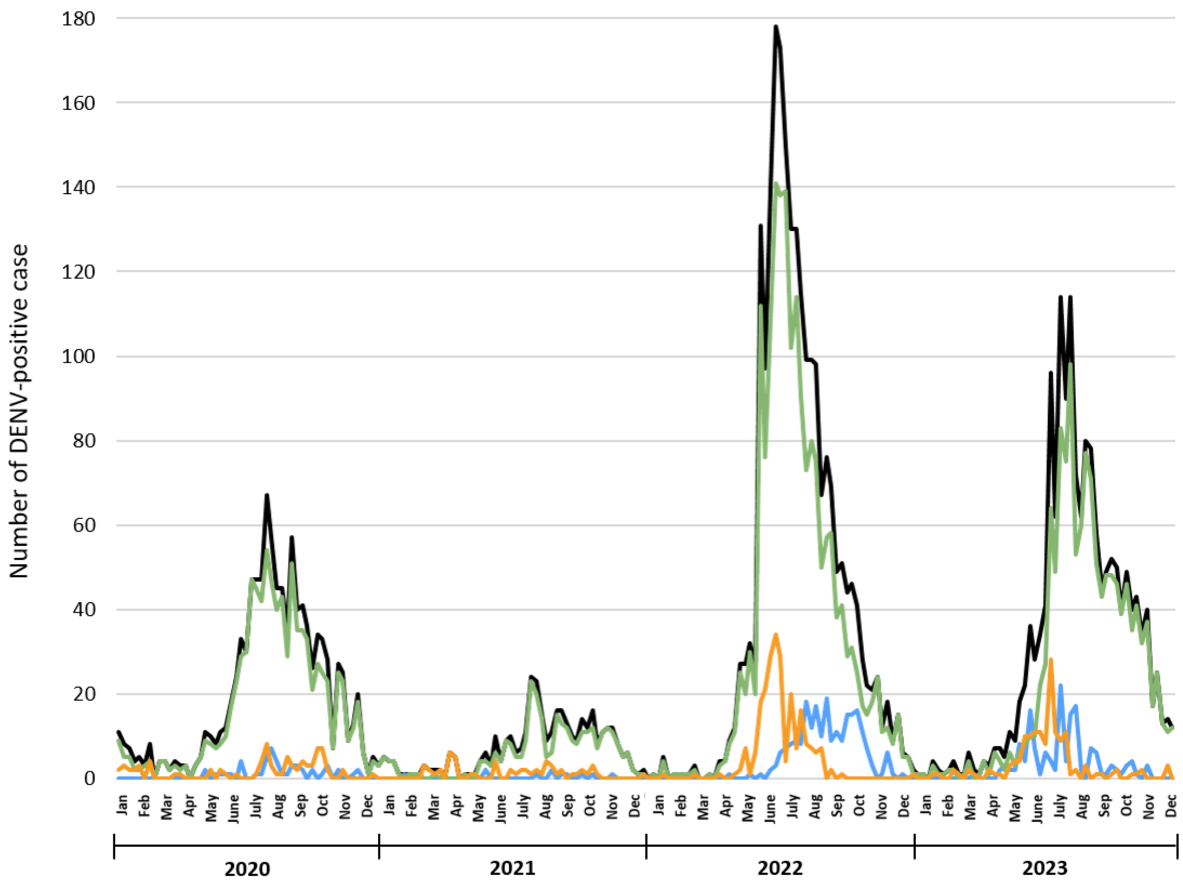

Figure S1. Number of DENV-positive cases reported monthly between 2020 and 2023. The same color code, as defined in Figure 1, is used to represent the origin of the DENV-positive cases (blue = North, green = Central, orange = South), and the black line represents the total sum (North, Central, and South regions) of DENV-positive cases reported in this study.
