# Supplementary material for: Molecular Epidemiology of Dengue Viruses in Lao People’s Democratic Republic, 2020–2023
Source: Microorganisms. 2025 Feb 1;13(2):318. doi: 10.3390/microorganisms13020318 (PMC11857872; doi:10.3390/microorganisms13020318)
Supplement: Supplementary file 1 [file microorganisms-13-00318-s001.zip › TROUPIN-FigureS2.pdf]

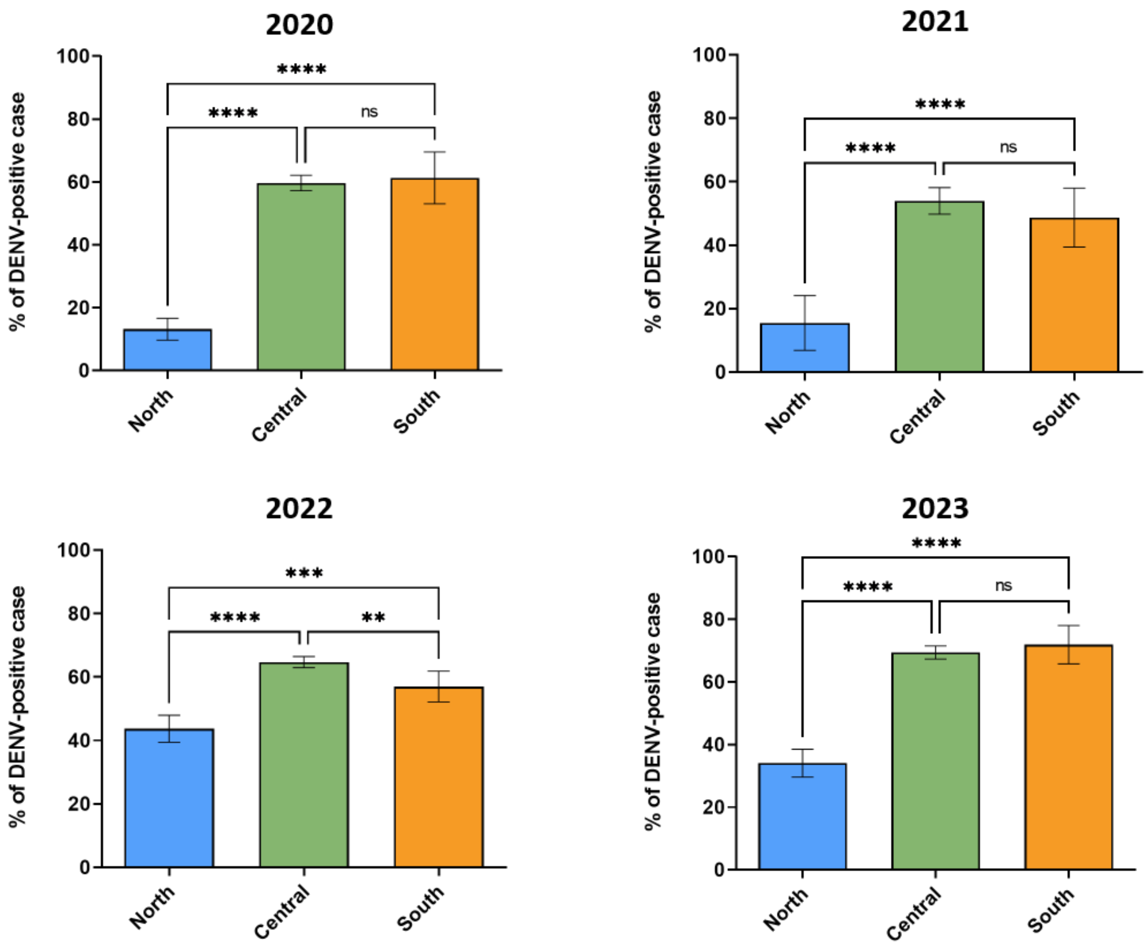

Figure S2. Percentage of DENV-positive cases according to geographical origin in years 2020-2023. The same color code, as defined in Figure 1, is used to represent the origin of the DENV-positive cases (blue = North, green = Central, orange = South). The error bars indicate 95% confidence intervals. The p-values are represented as follows: ns = non-significant, ** = < 0,01, *** = <0.001, **** = <0.0001.
