# Supplementary material for: Molecular Epidemiology of Dengue Viruses in Lao People’s Democratic Republic, 2020–2023
Source: Microorganisms. 2025 Feb 1;13(2):318. doi: 10.3390/microorganisms13020318 (PMC11857872; doi:10.3390/microorganisms13020318)
Supplement: Supplementary file 1 [file microorganisms-13-00318-s001.zip › TROUPIN-FigureS3.pdf]

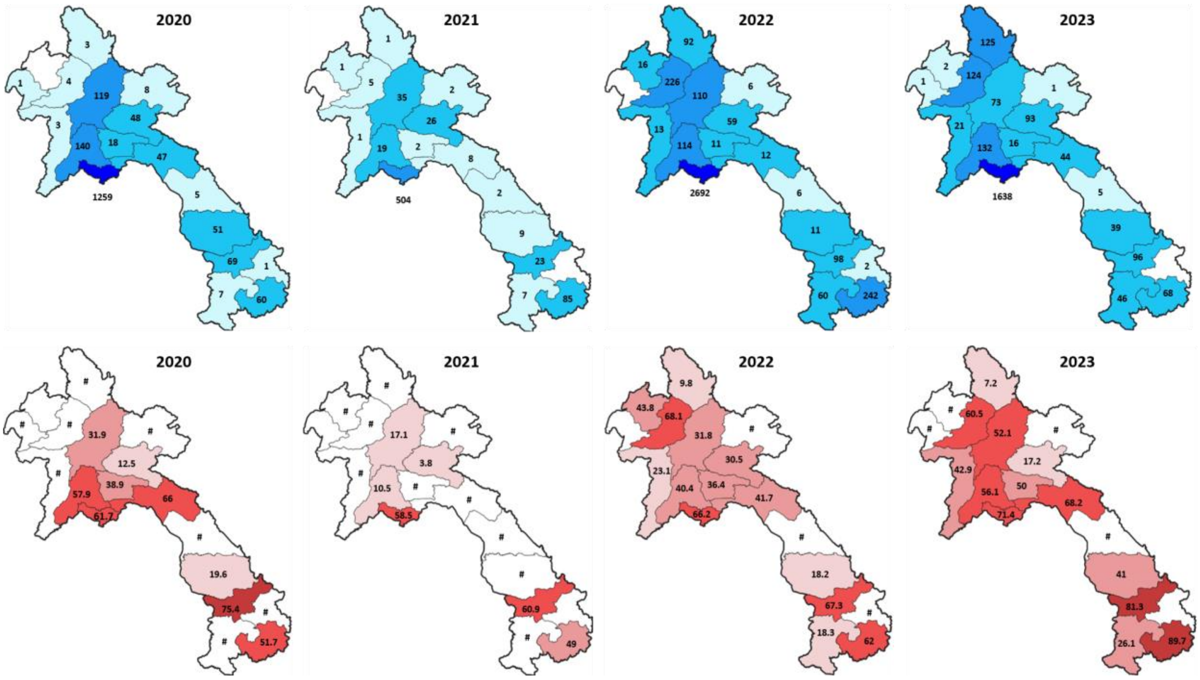

Figure S3. Geographical distribution of samples tested and DENV-positive cases in Lao PDR per year between 2020 and 2023. The blue-scale maps (top panels) show the total number of samples tested in this study by province and per year, with the total of each province indicated in black. Provinces in which no sample has been collected are in white. The red-scale maps (bottom panels) indicate the percentage of DENV-positive cases per province and year, with positivity rates shown in black. Provinces with fewer than 10 tested samples are represented in white and marked with a #.
