# Supplementary material for: Molecular Epidemiology of Dengue Viruses in Lao People’s Democratic Republic, 2020–2023
Source: Microorganisms. 2025 Feb 1;13(2):318. doi: 10.3390/microorganisms13020318 (PMC11857872; doi:10.3390/microorganisms13020318)
Supplement: Supplementary file 1 [file microorganisms-13-00318-s001.zip › TROUPIN-TableS1.pdf]

Table S1. List of the Lao DENV strains selected for sequencing and phylogenetic analysis.

Sample ID #	Province	Date of Collection	Serotype	Genotype	GenBank Accession Number
2020-16637	Vientiane Capital	26/07/2020	DENV-1	DENV-1I	PQ775559
2020-16848	Luangprabang	04/08/2020	DENV-1	DENV-1I	PQ775560
2020-16885	Attapeu	22/07/2020	DENV-1	DENV-1I	PQ775561
2020-16901	Vientiane Capital	15/08/2020	DENV-1	DENV-1I	PQ775562
2021-17912	Vientiane Capital	28/06/2021	DENV-1	DENV-1I	PQ775563
2021-17968	Vientiane Capital	22/07/2021	DENV-1	DENV-1I	PQ775564
2021-18036	Vientiane Capital	06/08/2021	DENV-1	DENV-1I	PQ775565
2021-18088	Huaphanh	23/08/2021	DENV-1	DENV-1I	PQ775566
2021-18116	Saravane	22/08/2021	DENV-1	DENV-1I	PQ775567
2021-18269	Champasack	06/10/2021	DENV-1	DENV-1I	PQ775568
2021-18421	Vientiane Capital	13/12/2021	DENV-1	DENV-1I	PQ775569
2022-19556	Attapeu	03/07/2022	DENV-1	DENV-1I	PQ775570
2022-19576	Phongsaly	23/06/2022	DENV-1	DENV-1I	PQ775571
2022-19598	Vientiane Capital	06/07/2022	DENV-1	DENV-1I	PQ775572
2022-19666	Vientiane Capital	09/07/2022	DENV-1	DENV-1I	PQ775573
2022-19889	Oudomxay	11/07/2022	DENV-1	DENV-1I	PQ775574
2022-19907	Xiengkhuang	04/07/2022	DENV-1	DENV-1I	PQ775575
2022-20118	Xaysomboune	30/06/2022	DENV-1	DENV-1I	PQ775576
2022-20132	Vientiane province	19/07/2022	DENV-1	DENV-1I	PQ775577
2022-20184	Luangnamtha	23/07/2022	DENV-1	DENV-1I	PQ775578
2022-20475	Vientiane Capital	04/08/2022	DENV-1	DENV-1I	PQ775579
2022-20561	Vientiane Capital	05/08/2022	DENV-1	DENV-1I	PQ775580
2022-20698	Champasack	03/08/2022	DENV-1	DENV-1I	PQ775581
2022-20930	Attapeu	20/07/2022	DENV-1	DENV-1I	PQ775582
2022-20992	Luangprabang	21/08/2022	DENV-1	DENV-1I	PQ775583
2022-21067	Vientiane Capital	25/08/2022	DENV-1	DENV-1I	PQ775584
2023-22315	Vientiane Capital	03/04/2023	DENV-1	DENV-1I	PQ775585
2023-22399	Attapeu	06/05/2023	DENV-1	DENV-1I	PQ775586
2023-22416	Phongsaly	12/05/2023	DENV-1	DENV-1I	PQ775587
2023-22535	Saravane	11/06/2023	DENV-1	DENV-1I	PQ775588
2023-22580	Savannakhet	20/06/2023	DENV-1	DENV-1I	PQ775589
2023-22631	Oudomxay	18/06/2023	DENV-1	DENV-1I	PQ775590
2023-22926	Vientiane Capital	23/07/2023	DENV-1	DENV-1I	PQ775591
2023-22995	Attapeu	17/07/2023	DENV-1	DENV-1I	PQ775592
2023-23357	Xiengkhuang	24/07/2023	DENV-1	DENV-1I	PQ775593
2023-23448	Attapeu	29/07/2023	DENV-1	DENV-1I	PQ775594
2023-23458	Oudomxay	09/08/2023	DENV-1	DENV-1I	PQ775595
2023-23488	Vientiane Capital	19/08/2023	DENV-1	DENV-1I	PQ775596
2023-23679	Oudomxay	20/08/2023	DENV-1	DENV-1I	PQ775597
2023-24243	Luangprabang	12/09/2023	DENV-1	DENV-1I	PQ775598
2023-24374	Savannakhet	01/11/2023	DENV-1	DENV-1I	PQ775599
2023-24580	Vientiane Capital	24/11/2023	DENV-1	DENV-1I	PQ775600

2020-16011	Vientiane Capital	13/02/2020	DENV-2	DENV-2II	PQ775601
2020-16253	Vientiane province	18/06/2020	DENV-2	DENV-2II	PQ775602
2020-16323	Savannakhet	24/06/2020	DENV-2	DENV-2II	PQ775603
2020-16409	Vientiane province	08/07/2020	DENV-2	DENV-2II	PQ775604
2020-17110	Vientiane Capital	02/09/2020	DENV-2	DENV-2II	PQ775605
2020-17258	Saravane	17/09/2020	DENV-2	DENV-2II	PQ775606
2020-17445	Luangprabang	30/09/2020	DENV-2	DENV-2II	PQ775607
2020-17559	Vientiane Capital	12/11/2020	DENV-2	DENV-2II	PQ775608
2021-17728	Vientiane Capital	18/01/2021	DENV-2	DENV-2II	PQ775609
2021-17782	Saravane	27/08/2020	DENV-2	DENV-2II	PQ775610
2021-17934	Vientiane Capital	09/07/2021	DENV-2	DENV-2II	PQ775611
2021-18019	Vientiane Capital	02/08/2021	DENV-2	DENV-2II	PQ775612
2021-18041	Vientiane Capital	08/08/2021	DENV-2	DENV-2II	PQ775613
2021-18049	Luangprabang	05/08/2021	DENV-2	DENV-2II	PQ775614
2021-18240	Vientiane Capital	28/09/2021	DENV-2	DENV-2II	PQ775615
2021-18374	Vientiane Capital	12/11/2021	DENV-2	DENV-2II	PQ775616
2022-20125	Savannakhet	19/07/2022	DENV-2	DENV-2II	PQ775617
2022-20147	Champasack	23/06/2022	DENV-2	DENV-2II	PQ775618
2022-20364	Vientiane Capital	30/07/2022	DENV-2	DENV-2II	PQ775619
2022-20437	Oudomxay	26/07/2022	DENV-2	DENV-2II	PQ775620
2022-20684	Luangprabang	09/08/2022	DENV-2	DENV-2II	PQ775621
2022-20847	Vientiane Capital	10/08/2022	DENV-2	DENV-2II	PQ775622
2022-20974	Attapeu	02/08/2022	DENV-2	DENV-2II	PQ775623
2022-21088	Attapeu	19/08/2022	DENV-2	DENV-2II	PQ775624
2023-22347	Vientiane Capital	22/04/2023	DENV-2	DENV-2II	PQ775625
2023-22537	Saravane	15/06/2023	DENV-2	DENV-2II	PQ775626
2023-22572	Oudomxay	17/06/2023	DENV-2	DENV-2II	PQ775627
2023-22622	Vientiane Capital	29/06/2023	DENV-2	DENV-2II	PQ775628
2023-22670	Saravane	26/06/2023	DENV-2	DENV-2II	PQ775629
2023-22812	Vientiane Capital	11/07/2023	DENV-2	DENV-2II	PQ775630
2023-23165	Vientiane Capital	02/08/2023	DENV-2	DENV-2II	PQ775631
2023-23554	Savannakhet	23/08/2023	DENV-2	DENV-2II	PQ775632
2023-23556	Vientiane Capital	23/08/2023	DENV-2	DENV-2II	PQ775633
2023-23650	Champasack	31/08/2023	DENV-2	DENV-2II	PQ775634
2023-24130	Vientiane Capital	11/10/2023	DENV-2	DENV-2II	PQ775635
2023-24159	Oudomxay	05/10/2023	DENV-2	DENV-2II	PQ775636
2023-24342	Vientiane Capital	31/10/2023	DENV-2	DENV-2II	PQ775637
2023-24384	Luangnamtha	01/11/2023	DENV-2	DENV-2II	PQ775638
2023-24608	Xiengkhuang	22/11/2023	DENV-2	DENV-2II	PQ775639
2023-24657	Vientiane Capital	07/12/2023	DENV-2	DENV-2II	PQ775640
2020-16064	Vientiane Capital	24/03/2020	DENV-4	DENV-4I	PQ775641
2020-16191	Vientiane Capital	02/06/2020	DENV-4	DENV-4I	PQ775642
2020-16235	Vientiane Capital	11/06/2020	DENV-4	DENV-4I	PQ775643
2020-16245	Vientiane Capital	12/06/2020	DENV-4	DENV-4I	PQ775644
2020-16248	Vientiane Capital	15/06/2020	DENV-4	DENV-4I	PQ775645
2020-16288	Vientiane Capital	19/06/2020	DENV-4	DENV-4I	PQ775646

2020-16332	Vientiane Capital	27/06/2020	DENV-4	DENV-4I	PQ775647
2020-16420	Vientiane Capital	09/07/2020	DENV-4	DENV-4I	PQ775648
2020-16743	Vientiane Capital	03/08/2020	DENV-4	DENV-4I	PQ775649
2020-16831	Vientiane Capital	10/08/2020	DENV-4	DENV-4I	PQ775650
2020-16833	Vientiane Capital	10/08/2020	DENV-4	DENV-4I	PQ775651
2020-16949	Vientiane Capital	18/08/2020	DENV-4	DENV-4I	PQ775652
2020-17142	Vientiane Capital	09/09/2020	DENV-4	DENV-4I	PQ775653
2020-17143	Vientiane Capital	09/09/2020	DENV-4	DENV-4I	PQ775654
2020-17296	Vientiane Capital	25/09/2020	DENV-4	DENV-4I	PQ775655
2020-17322	Vientiane Capital	30/09/2020	DENV-4	DENV-4I	PQ775656
2020-17329	Vientiane Capital	01/10/2020	DENV-4	DENV-4I	PQ775657
2020-17428	Vientiane Capital	14/10/2020	DENV-4	DENV-4I	PQ775658
2021-17754	Vientiane Capital	12/02/2021	DENV-4	DENV-4I	PQ775659

#: Sample ID, defined by the year of reception and the order of receipt at the laboratory.
